# Differential expression of *N*-linked oligosaccharides in methotrexate-resistant primary central nervous system lymphoma cells

**DOI:** 10.1186/s12885-019-6129-8

**Published:** 2019-09-11

**Authors:** Yasuo Takashima, Takeshi Yoshimura, Yuichiro Kano, Azusa Hayano, Hiroaki Hondoh, Kazuhiro Ikenaka, Ryuya Yamanaka

**Affiliations:** 1 Laboratory of Molecular Target Therapy for Cancer, Graduate School of Medical Science, Kyoto Prefectural University of Medicine, 465 Kajii-cho, Kawaramachi-Hirokoji, Kamigyo-ku, Kyoto, 602-8566 Japan; 2 Division of Neurobiology and Bioinformatics, National Institute for Physiological Sciences, National Institutes of Natural Sciences, Okazaki, Aichi 444-8787 Japan; 3 Present Address: Department of Child Development and Molecular Brain Science, United Graduate School of Child Development, Osaka University, Suita, Osaka 565-0871 Japan; 4 Department of Neurosurgery, Toyama Prefectural Central Hospital, Toyama, 930-8550 Japan

**Keywords:** Primary central nervous system lymphoma, Diffuse large B-cell lymphoma, Methotrexate, Sialylated *N*-linked oligosaccharide, High performance liquid chromatography

## Abstract

**Background:**

Oligosaccharides of glycoprotein, particularly negatively-charged sialylated *N*-glycans, on the surface of lymphomas play important roles in cell–cell interactions and bind immunoglobulin-like lectins, causing inflammatory responses and bioregulation. However, their characterizations have largely been unknown in central nervous system (CNS) lymphoma.

**Methods:**

Here, we investigated expression patterns of *N*-linked oligosaccharides of glycoproteins in cells derived from CNS lymphomas and clinical specimens.

**Results:**

We first generated methotrexate (MTX)-resistant cells derived from HKBML and TK as CNS lymphoma, and RAJI as non-CNS lymphoma and determined *N*-linked oligosaccharide structures in these cells and other non-CNS lymphoma-derived cells including A4/FUK, OYB, and HBL1. Major components of the total oligosaccharides were high-mannose type *N*-glycans, whose level increased in MTX-resistant HKBML and TK but decreased in MTX-resistant RAJI. We also detected sialylated biantennary galactosylated *N*-glycans with α1,6-fucosylation, A2G2F, and A2G2FB from HKBML, TK, and RAJI. Sialylated A4G4F was specifically isolated from RAJI. However, the ratios of these sialylated *N*-glycans slightly decreased against MTX-resistant compared to non-resistant cells. Interestingly, almost all complex-type oligosaccharides were α2,6-sialylated.

**Discussion:**

This is the first study for the expression profile of *N*-oligosaccharides on MTX-resistant primary CNS lymphoma-derived cells HKBML and TK, and tumor tissues resected from patients with CNS lymphoma,

**Conclusion:**

These results propose a possibility that the differential expression of high-mannose types and sialylated A2G2F, A2G2FB, and A4G4F on the surface of CNS lymphomas may provide a hint for targets for diagnoses and treatments of the oligosaccharide type-specific lymphomas.

## Background

Oligosaccharides are saccharide polymers that have various functions such as cell–cell interactions and cell recognitions, including immune responses [1]. Glycans are normally formed by presenting sugar chains linked to lipids or amino acid side chains by *N*- or *O*-glycosidic bonds [2]. The *O*-linked oligosaccharide is attached to threonine (Thr) or serine (Ser) [3], while the *N*-linked oligosaccharide is generally pentasaccharides attached to asparagine (Asn) by β-linkage to amine nitrogen of side chains [4]. The *N*-oligosaccharides of glycoproteins show differential patterns of branching formations by *N*-acetylglucosaminyltransferase activities at *N*-acetylglucosamine (GlcNAc) residues in types of Pl-2, Pl-4, and B1 + 6 linkage to mannose (Man) residues of the core [5, 6]. Previous studies have also demonstrated the mechanisms of intracellular trafficking of the cell surface of glycoproteins and their subsequent returns to the cell surface [7–9].

Several studies have clarified that the structures of *N*-linked oligosaccharide chains on glycoproteins have been involved in tumor cell adhesion to the extracellular matrix (ECM), metastatic potentials, and cell proliferation and differentiation [8, 10–15]. Metastatic potentials of tumor cells have been shown to correlate with the expression of highly branched tri- and tetra-antennary β-1,6-GlcNAc-bearing *N*-glycans [16, 17]. The increased β1,6-GlcNAc-bearing *N*-glycan expression is co-regulated by *N*-acetyl glucosaminyl transferases V (GnT-V) and the Ets-1 transcription factor, and the branching complex type *N*-glycans function in glioma invasivity [16]. *N*-acetyl glycan structures and physicochemical properties regulate cell proliferation and differentiation in leukemia [12]. A semi-automated systematic detection system for analyzing the *N*-linked oligosaccharides of glycoproteins has been developed [18, 19]. *N*-linked oligosaccharides are relatively easily detectable from a small amount of acetone-precipitated sample (e.g., 1–2 mg) [20]. The oligosaccharides on the cell surface of gliomas are well-examined in T cell immune responses and sensitivity to killer lymphocytes [21–23]. Several studies clarify a strong correlation between the lectin-binding and the biological function in diffuse large B-cell lymphoma (DLBCL) [24–28]. However, the oligosaccharides on the primary central nervous system (CNS) lymphoma (PCNSL) surface have largely been unknown.

PCNSL is a rare subtype of DLBCL, which is an aggressive variant of extra-nodal non-Hodgkin’s lymphoma (NHL) [29]. PCNSL only accounts for 3% of primary CNS tumors and 1% of NHLs in adults [30]. Methotrexate (MTX) is an antifolate that inhibits the dihydrofolate reductase activity in purine and thymidine syntheses and regulates the expression of glucocorticoid receptor α and β in human blood cells in vitro [31, 32]. High-dose methotrexate (HD-MTX) is used as a first-line treatment in PCNSL [33]. Moreover, second-line treatments are also required for 10–35% of patients with refractory diseases and for another 35–60% or more who have relapse-acquired resistances [34]. Eventually, although treatment with HD-MTX is used as a standard treatment in PCNSL, most of the cases come to relapse-acquired resistances to MTX [35].

Here, we generated MTX-resistant lymphoma cell lines derived from PCNSL and non-CNS lymphoma, which were applied to the semi-automated detection system for the *N*-linked oligosaccharides of glycoproteins on the cell surfaces by using *N*-pyridylamination fluorescent labels and reverse- and normal-phase high performance liquid chromatography (HPLC), in addition to CNS lymphoma specimens derived from the patients. Consequently, we obtained the results for differential expression patterns of *N*-oligosaccharides among PCNSL-derived cells, non-CNS lymphoma-derived cells, and clinical specimens of CNS lymphoma and for the slightly decreased expression in MTX-resistant lymphomas, including human brain malignant lymphomas HKBML and TK, and non-CNS lymphoma RAJI, which was seemed those in CNS lymphomas. This study is the first report for the expression patterns of *N*-oligosaccharides on tumor tissues resected from patients with CNS lymphomas and MTX-resistant PCNSL-derived cells, including HKBML and TK. The results may be a hint for understanding the status and microenvironments of the surface glycans of lymphomas and may be useful for development of applied target therapies for cell recognitions.

## Methods

### Clinical specimens

Primary and secondary CNS lymphomas, which were pathologically diagnosed DLBCL tissue specimens, were obtained from Toyama Prefectural Central Hospital (Additional file 1: Table S1). All study protocols were approved by both of the Institutional Review Boards of Toyama Prefectural Central Hospital and Kyoto Prefectural University of Medicine (approval number 2011–1081), and experiments were performed in accordance with institutional guidelines. Written informed consents were obtained from all patients. Resected tumor tissues were immediately snap-frozen and fixed in 4% (v/v) paraformaldehyde (PFA) for 24 h, and then substituted with phosphate-buffered saline (PBS).

### Cells

A4/FUK [36] and TK [37] were purchased from JCRB Cell Bank (NIBIOHN: National Institutes of Biomedical Innovation, Health and Nutrition) (Additional file 1: Table S1). HKBML [38] and RAJI [39] were purchased from RIKEN Cell Bank (RIKEN BRC: RIKEN BioResource Center) (Additional file 1: Table S1). HBL1 [40] and OYB [41] were distributed from Kyoto University (Additional file 1: Table S1). Cells were grown according to standard protocol in Ham’s F12 medium (Nacalai Tesque) with 15% fetal bovine serum (FBS) (Thermo Fisher Scientific) for HKBML, Roswell Park Memorial Institute (RPMI) 1640 medium (Nacalai Tesque) with 20% FBS for TK, and RPMI 1640 medium with 10% FBS for A4/FUK, HBL1, OYB, and RAJI in 5% CO_2_ at 37 °C. MTX-resistant cells were generated as shown in Additional file 2: Figure S1A.

### *N*-oligosaccharides analysis

Purification and pyridylamination of oligosaccharides were performed as described [19, 42, 43]. Pyridylated (PA)-*N*-oligosaccharides were separated into neutral *N*-oligosaccharides and mono−/di−/tri−/tetra-sialylated *N*-oligosaccharides through an anion-exchange column (Mono Q5/50GL, GE Healthcare) using HPLC and DE52-packed column (Whatman, GE Healthcare) [44]. Sialylated PA-*N*-glycans were treated with neuraminidase at 37 °C for 14 h in 50 mM ammonium acetate (pH 5.0) to cleave sialic acids, followed by heating at 100 °C for 5 min and filtering through 0.2 μm spin filter (Ultrafree-MC LG, Millipore). Neutral PA-*N*-oligosaccharides were analyzed using HPLC as described [19, 42, 43]. *N*-oligosaccharide structures were determined by calculating the mannose unit value from NP-HPLC (Takara Bio) and the glucose unit value from RP-HPLC (Takara Bio), as described [20, 45], also by comparison with known standards and sequential exoglycosidase digestion (see below). PA-*N*-glycans were quantified as described [44], and HPLC chromatogram data were analyzed using Unipoint (Gilson), LC station (Shimadzu), and Empower2 (Waters). The workflow of *N*-oligosaccharides analysis and separation by HPLC is shown in Additional file 2: Figure S1B.

### Exoglycosidase digestion

Exoglycosidase digestion was performed as described [44]. In brief, purified PA-*N*-oligosaccharides were digested for 3 h at 37 °C using the following enzymes: *Xanthomonas manihotis* β1,3-galactosidase (New England BioLabs) for β (1–3)-Gal in 50 mM sodium acetate (pH 4.5) with 100 μg/mL bovine serum albumin (BSA); *Diplococcus pneumoniae* β-galactosidase (Roche Diagnostics) for β1,4-Gal in 50 mM sodium acetate (pH 6.0); α1,3/4-L-fucosidase (Takara Bio) for α1,3/4Fuc in 100 mM sodium phosphate buffer (pH 6.0); bovine kidney α1,6-fucosidase (ProZyme) for α1–6 > 1–2/3/4-Fuc in 100 mM sodium phosphate buffer (pH 6.0). PA-*N*-oligosaccharides were also digested for 14 h at 37 °C by neuraminidase for α2,3/6/8-NeuAc in 50 mM ammonium acetate (pH 5.0) and α2,3-sialidase for α (2, 3) NeuAc in 50 mM sodium citrate (pH 6.0) with 100 mM NaCl and 100 μg/mL BSA.

### Matrix assisted laser desorption ionization time-of-flight mass spectrometry (MALDI/TOF-MS)

Molecular masses of PA-sugar chains and their isobaric monosaccharide compositions were determined by MALDI/TOF-MS as described [44]. One microliter of matrix solution of 10 mg/mL 2,5-dihydroxybenzoic acid in 30% acetonitrile was spotted on the plate, 1 μL of sample solution was added and then dried by warm air. MALDI/TOF mass spectra were acquired using REFLEX mass spectrometer (Bruker-Franzen) in the positive and reflector mode at an acceleration voltage of 20 kV and delayed ion extraction. Standard PA-oligosaccharides were used to achieve a two-point external calibration for mass assignment of ions. The mass spectra shown were the sum of at least 30 laser shots.

### Heatmap analysis

Heatmaps were constructed by color-coding standardized log or linear scales, indicating fold-differences of neutral and sialylated sugar chains in MTX-resistant cells compared to corresponding control cells, or by using the percent (%) area of fractionated peaks, designated by peak numbers, eluting at different retention times. Color configurations are shown in each panel. NA, not applicable.

### Statistics

Statistics were performed using JMPv10 built-in-modules (SAS Institute Inc., Tokyo, Japan). Principal component analysis was performed to estimate Pearson correlation. *P* < 0.05 was considered statistically significant.

## Results

### Generation of MTX-resistant lymphoma cells

**Fig. 1:**
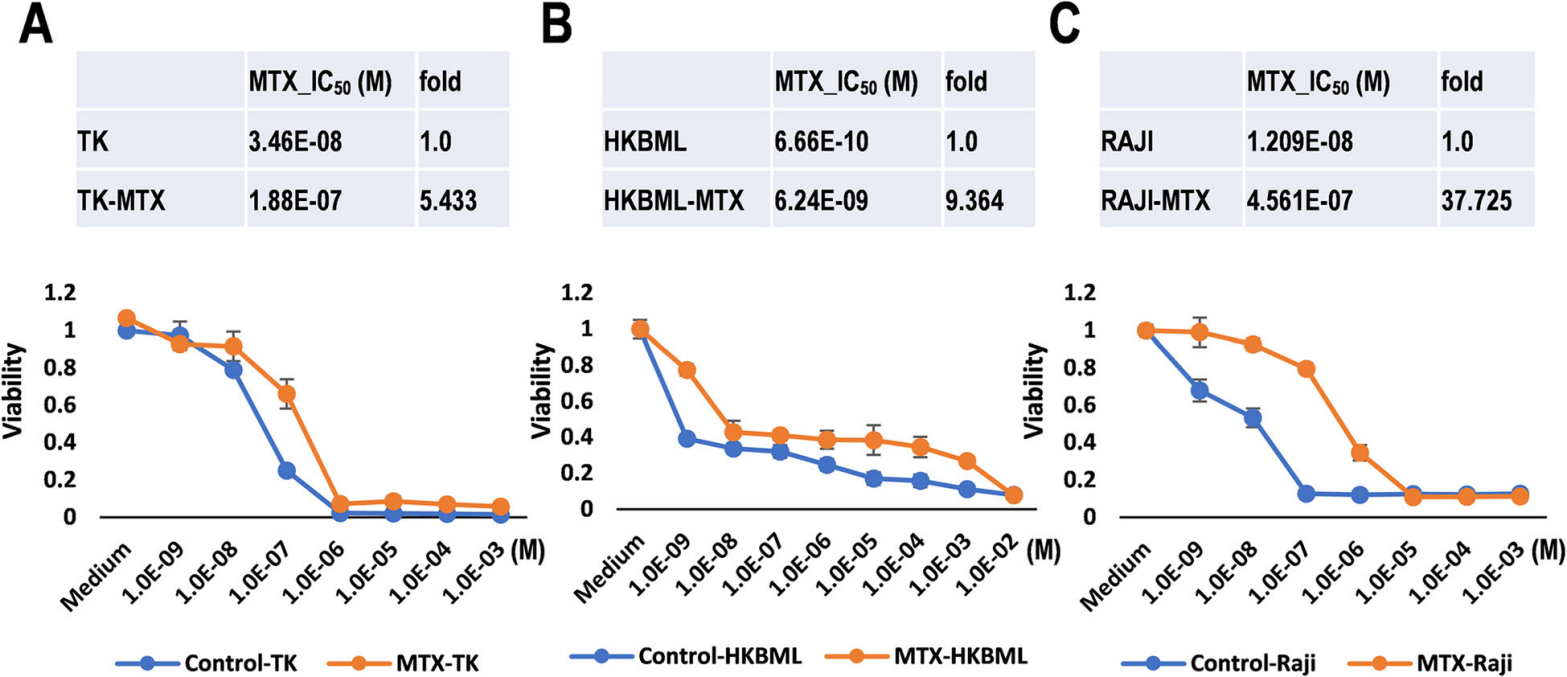
Generation of the MTX-resistant cell lines. WST-8 cell proliferation assays on the cells derived from TK, HKBML, and RAJI in appropriate culture media with diluted MTX from 1 × 10^− 2^ to 1 × 10^− 9^ mol m^− 3^ (M). **a** Control and MTX-resistant TK cells. **b** Control and MTX-resistant HKBML cells. **c** Control and MTX-resistant RAJI cells. Calculated IC_50_ and fold changes are shown on the top of graphs

The aim of this study is a comparison with *N*-glycan profiling between CNS lymphoma cells and their derived MTX-resistant cells and thereby an annotation clinical specimens of CNS lymphomas. First, to examine differential expression of oligosaccharides of glycoproteins on the surface of the cells, we generated MTX-resistant lymphoma cells (Fig. 1), including HKBML and TK as PCNSL, and RAJI as non-CNS lymphoma, in addition to HBL, OYB, and A4/FUK as other non-CNS lymphoma references (Additional file 1: Table S1, and Additional file 2: Figure S1A). HKBML was cultured with 1.0 × 10^− 7^ mol m^− 3^ (M) MTX for 6 weeks following pre-culture with lower concentrations (1.0 × 10^− 9^–1.0 × 10^− 8^ M) of MTX for 4 weeks. TK was cultured with 1.0 × 10^− 6^ M MTX for 6 weeks following pre-culture with lower concentrations (1.0 × 10^− 9^–1.0 × 10^− 7^ M) of MTX for 9 weeks. RAJI was cultured with 1.0 × 10^− 6^ M MTX for 6 weeks following pre-culture with lower concentrations (1.0 × 10^− 9^–1.0 × 10^− 7^ M) of MTX for 18 weeks. The resultant cells including MTX-resistant TK, MTX-resistant HKBML, and MTX-resistant RAJI obtained resistances against MTX with 5.43-fold (inhibitory concentration 50 (IC_50_) = 1.88 × 10^− 7^ M), 9.36-fold (IC_50_ = 6.24 × 10^− 9^ M), and 37.72-fold (IC_50_ = 4.56 × 10^− 7^ M), respectively (Fig. 1a–c), indicating that acquired resistances to MTX in PCNSL-derived TK and HKBML cells are weak than that in non-CNS lymphoma-derived RAJI cells. After collection of cells, samples were used to analyze the *N*-oligosaccharides of glycoproteins on the cell surface using pyridylamination fluolabeling and subsequent HPLC (Additional file 2: Figure S1B).

### Expression and structures of *N*-linked oligosaccharides in lymphoma cells

DE52 diethylaminoethyl cellulose (DEAE-C) anion-exchange column chromatography was performed using HKBML, TK, RAJI, OYB, HBL1, and A4/FUK (Additional file 3: Figure S2A and Additional file 4: Figure S3A), and MTX-resistant HKBML, TK, and RAJI (Additional file 3: Figure S2A). Coupled with standard unit for sialylated sugar chains, the peaks representing non-sialylated (S0) to 4-sialylated (S4) sugar chains were detected (Additional file 3: Figure S2A and Additional file 4: Figure S3A). The left-side peaks at the S1 peak indicated the *N*-oligosaccharides coupling with an unknown acidic group, and the left-side peaks at the S2 peak indicated the *N*-mono-sulfated oligosaccharides (Additional file 2: Figure S1A and Additional file 3: Figure S2A). Fractions eluting at wide-range retention times of S0 were further analyzed using normal-phase HPLC in neutral sugar chains and sialylated sugar chains (Additional file 3: Figures S2B–G and Additional file 4: Figure S3B, C). Fractions eluting at retention times of M4-M9 were collected and analyzed, and the oligostructures included in the peaks were determined by MALDI/TOF-MS, then referred to the oligosaccharide structures in Additional file 5: Figure S4. High mannose type oligosaccharides, including M4B, M5A, M6B, M7A, M7B, M8A, and M9A were determined with mannose unit standard in all samples examined (Additional file 3: Figure S2B–G, Additional file 4: Figure S3B, C, and Additional file 5: Figure S4). Interestingly, biantennary bigalactosylated structure with α1,6-fucosylation, A2G2F, and the *N*-acetylglucosamine-bisecting structure, A2G2FB as complex types, were also detected in neutral and sialylated oligosaccharides in cells derived from HKBML, TK, and RAJI (Additional file 3: Figure S2B–G and Additional file 5: Figure S4). Further, tetra-antennary tetra-galactosylated structure with α1,6-fucosylation, A4G4F, was only detected in cells from RAJI (Additional file 3: Figure S2F, G and Additional file 5: Figure S4). These complex types were not detected in HBL1, A4/FUK, and OYB (Additional file 4: Figure S3B, C).

### Differential expression patterns of *N*-linked oligosaccharides in MTX-resistant lymphoma cells

**Fig. 2:**
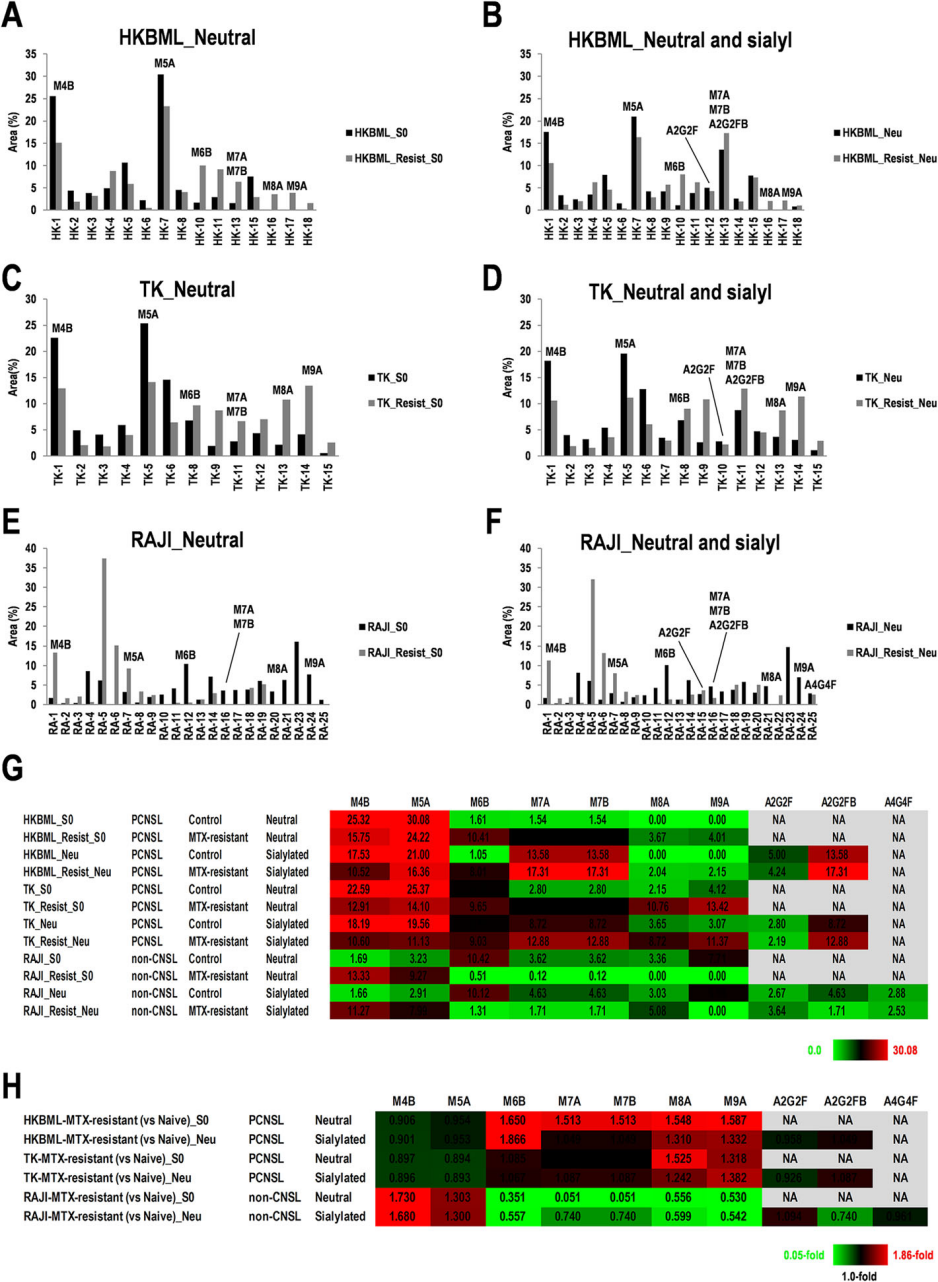
Expression changes of neutral and sialyl sugar chains in normal-phase HPLC analysis of the *N*-linked oligosaccharide patterns of human methotrexate (MTX)-resistant lymphoma cells. **a–f** Neutral sugar chain form HKBML (**a**), TK (**c**), and RAJI (**e**), and sialyl sugar chain from HKBML (**b**), TK (**d**), and RAJI (**f**) were examined. Intensities were evaluated with the percent (%) area of peak in fractions eluting at retention times of M4, M5, M6, M7, M8, and M9. The peak numbers refer to the oligosaccharide structures in Additional file 5: Figure S4. **g** Summary for expression changes of neutral and sialyl sugar chains among HKBML and TK as PCNSL, and RAJI as non-CNS lymphoma. Color configuration indicates high (red) to low (green). S0: non-sialyl sugar chain, Neu: Neuraminidase-treated sugar chains. **h** Summary for expression changes of sialyl sugar chains among HKBML and TK as PCNSL, and RAJI as non-CNS lymphoma. Fold changes of sialyl sugar chains from MTX-resistant cells compared to the corresponding non-resistant cells, including HKBML, TK, and RAJI. Color configuration indicates high (red) to low (green). NA, not applicable. S0: non-sialyl sugar chain, Neu: Neuraminidase-treated sugar chains

The ratios of peaks fractionating at appropriate retention time were calculated by converting to percent (%) area of the peaks in each cell (Fig. 2a–f and Additional file 6: Figure S5A, B), indicating almost expression ratios of distinct oligosaccharides of glycoproteins on surfaces of each cell. Expression ratios of each oligosaccharide were shown in a heat map as neutral sugar chains and neutral and sialylated sugar chains in non-resistant (control) and resistant cells for MTX (Fig. 2g and Additional file 6: Figure S5C). M4B, M5A, and M6B were highly expressed in PCNSL and non-CNS lymphoma derived cells, and expression levels of M7A/B, M8A, and M9A were relatively low. Further, sialylated A2G2F and A2G2FB were specifically detected in HKBML, TK, and RAJI. Besides, sialylated A4G4F was only detected in RAJI. Focusing on the differential expression of oligosaccharides in MTX-resistant cells compared to non-resistant cells derived from HKBML, TK, and RAJI, expression of M4B and M5A was increased in MTX-resistant RAJI (1.3–1.73-fold in log scale); M6B, M7A, M7B, M8A, and M9A were increased in MTX-resistant HKBML and TK (1.0–1.86-fold in log scale); however, expression changes between neutral sugar chains and neutral and sialylated sugar chains were irregular (0.69–14.57-fold in log scale) (Fig. 2h). Ectopic fold-expression of A2G2F and A2G2FB were also observed, whereas slightly changes in MTX-resistant HKBML, TK, and RAJI were observed compared to each corresponding control cell in neutral and sialylated sugar chains (0.74–1.09-fold in log scale) (Fig. 2h). A4G4F was only detected in MTX-resistant RAJI in neutral and sialylated sugar chains, but almost no changes were detected compared to the corresponding control cells (0.96-fold in log scale) (Fig. 2h). These results suggest that high-mannose types M5A and M6B may be possible targets for specific cells in PCNSL and non-CNS lymphoma. Besides, differential expression of sialylated A2G2F, A2G2FB, and A4G4F may be used to distinguish PCNSL and non-CNS lymphoma cells as a landmark in MTX-resistant lymphomas. However, the landmarks may function effectively, because there are almost no differences between the MTX-resistant and non-resistant cells in sialylated A2G2F, A2G2FB, and A4G4F.

### Sialylated *N*-linked oligosaccharides of glycoproteins on the cell surface of lymphomas

**Fig. 3:**
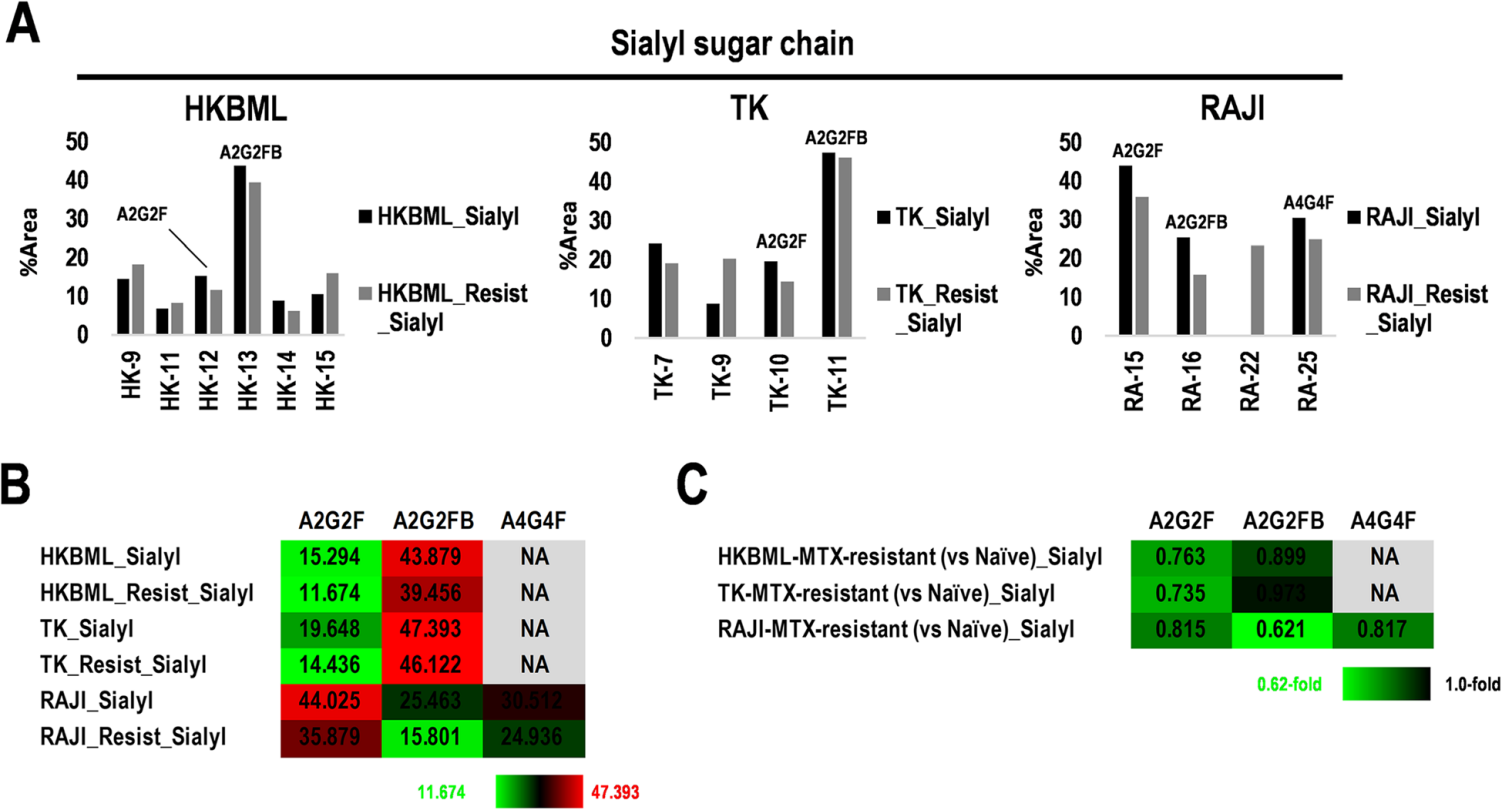
Differential expression of sialyl sugar chains in the *N*-linked oligosaccharide patterns of human methotrexate (MTX)-resistant lymphoma cells. **a** Percent area (%) of peak in fractions eluting at retention times of sialyl sugar chains, including A2G2F, A2G2FB, and A4G4F, form HKBML (left), TK (center), and RAJI (right) cells. **b** Summary of percent area of sialyl sugar chains among HKBML, TK, and RAJI, derived from A. Ratio (%) of sialyl sugar chains in cells, including MTX-resistant and non-resistant cells. Color configuration indicates high (red) to low (green). NA, not applicable. **c** Fold changes of sialyl sugar chains in MTX-resistant cells compared to the corresponding non-resistant cells, in HKBML, TK, and RAJI. Color configuration indicates 1.0-fold (black) to decrease (green). NA, not applicable

Calculating ratios of the peaks of neutral and/or sialylated sugar chains successfully returned the ratios of sialylated sugar chains (Fig. 3a, b and Additional file 6: Figure S5D, E). Interestingly, as for sialylated oligosaccharides, expression of A2G2F and A2G2FB were low and high in HKBML and TK as PCNSL, and RAJI as non-CNS lymphoma, respectively, whereas almost no change between MTX-resistant and non-resistant cells was observed (Fig. 3b). Sialylated A4G4F was only detected in RAJI (Fig. 3b) and slightly decreased by 0.81-fold in MTX-resistant RAJI compared to the non-resistant RAJI (Fig. 3c). However, fold-differences of complex type sialylated oligosaccharides detected in MTX-resistant HKBML, TK, and RAJI slightly decreased compared to the corresponding control cells (0.62–0.97-fold) (Fig. 3c). In non-CNS lymphoma cells, including A4/FUK and OYB, M7A and M7B were expressed, and especially highly expressed in OYB (41.5%) (Additional file 6: Figure S5D, E). A2G2F, A2G2FB, and A4G4F were hard to detected in A4/FUK, HBL1, and OYB (Additional file 6: Figure S5D, E). Although expression of A2G2F, A2G2FB, and A4G4F almost did not change in neutral and sialylated sugar chains, as described above (Fig. 2h), the results in only sialylated sugar chains indicated that the expression slightly decreased in MTX-resistant cells compared to the corresponding control cells, suggesting a careful attention for the strategies targeting sugar chains on the surface of MTX-resistant lymphomas. The ectopic expression of M7A and M7B was detected in OYB, which may be useful for marking distinct OYB-type non-CNS lymphoma from other types of lymphomas.

### Sialylated *N*-linked oligosaccharides of glycoproteins in clinical specimens of CNS lymphoma

**Fig. 4:**
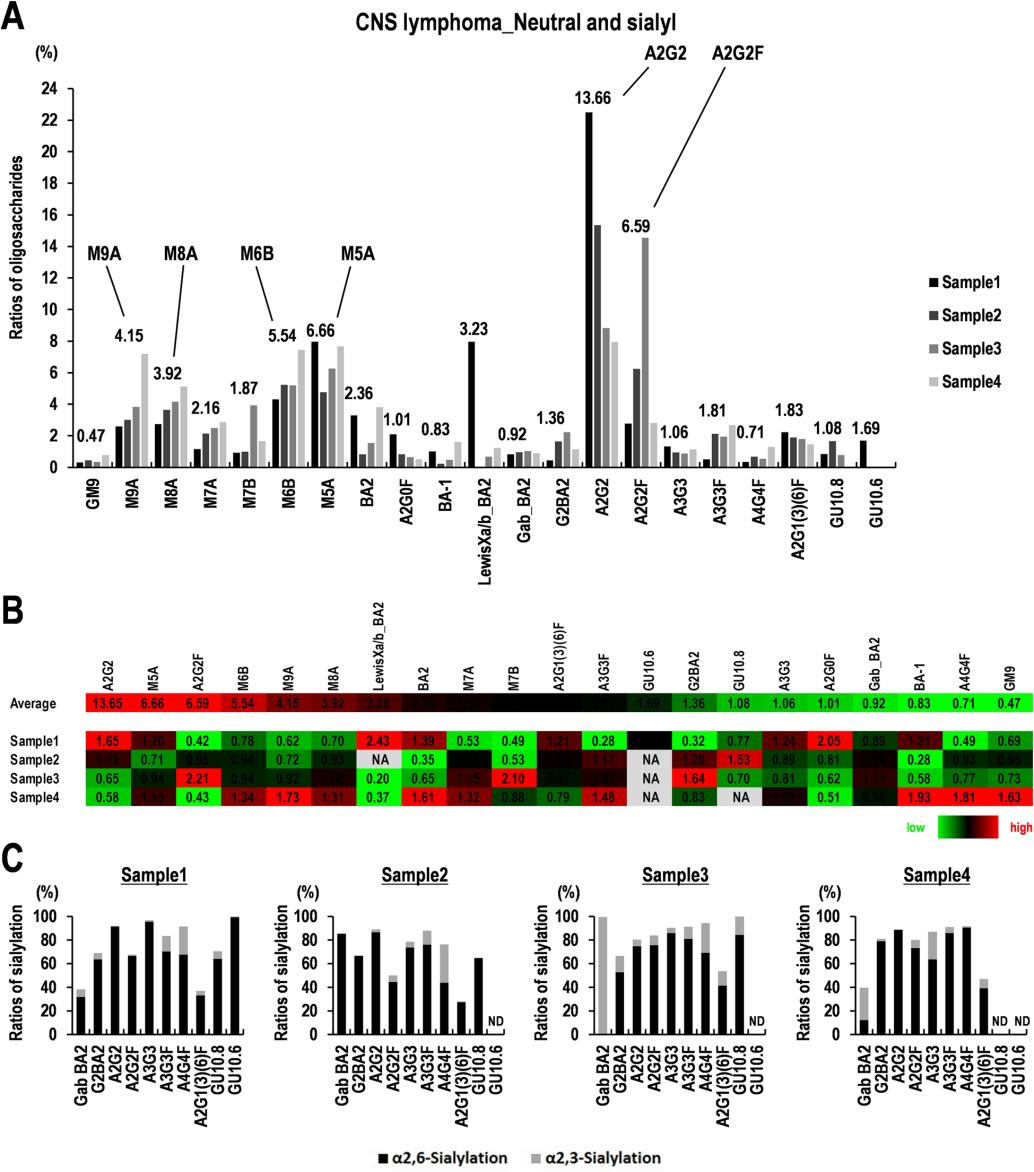
Expression of the sialylated oligosaccharides of *N*-glycoproteins in the clinical specimens of primary and secondary central nervous system (CNS) lymphomas. **a** Oligosaccharide types of *N*-glycoproteins. Ratios of oligosaccharides in each specimen are shown. The numbers in the graph represent the average ratios of oligosaccharides of four samples. **b** Relative expression of neutral and sialylated oligosaccharides of *N*-glycoproteins in the four clinical specimens. Average expression of *N*-oligosaccharides and their relative expression among the specimens are shown. Color configuration indicates high (red) to low (green). NA; not applicable. **c** Sialylation of oligosaccharides of *N*-glycoproteins. Ratios of α2,3- and α2,6-sialylation detected are shown

We further investigated the expression of *N*-linked oligosaccharides of glycoprotein in tumor tissues derived from three primary and one secondary CNS lymphoma specimens (Additional file 1: Table S1), and then detected 53–67% of the total oligosaccharides in the specimens (Fig. 4a). Relatively strong expression of A2G2, A2G2F, and LewisXa/b_BA2 as complex types and M5A, M6B, M8A, and M9A as high mannose types were detected in the four clinical specimens derived from CNS lymphomas (average ratios 3.28–13.65%) (Fig. 4b). Especially, the relatively strong expression in the secondary CNS lymphoma (Sample 4) was observed, compared with that in the PCNSL (Sample 1–3), for M6B (1.34-fold), M8A (1.31-fold), and M9A (1.73-fold) in strong expression (average ratios 3.92–5.54%), and BA-1 (1.93-fold), A4G4F (1.81-fold), and GM9 (1.63-fold) in weak expression (average ratios 0.83–0.47%) (Fig. 4b). Furthermore, 76.0 ± 19.8% of oligosaccharides were sialylated (Fig. 4c). Sialylation manners were α2,6-sialylation (62–68%) and α2,3-sialylation (6–21%) in CNS lymphoma specimens (Fig. 4c). Especially, M5A, M6B, M8A, M9A, A2G2, and A2G2F were also relatively highly expressed in the specimens (Fig. 4a, b). Of these, 50–90% of A2G2 and A2G2F were α2,6-sialylated (Fig. 4c). A2G2FB (also known as G2-BA-2) was detected in HKBML and TK cells (13.58 and 6.72%, respectively) (Fig. 2g), but hardly detected in the specimens (average ratio: 1.36%) (Fig. 4a). In addition, A2G2FB was α2,6-sialylated at 70–80% in the specimens (Fig. 4c). Interestingly, A4G4F was not detected in HKBML and TK cells and also little detected (0.7%) in the specimens (Fig. 4a). Compared to the results from the tumor specimens and cell lines, the high mannose types, including M5A, M6B, M7A, M7B, M8A, and M9A, in CNS lymphoma specimens, were expressed as well as HKBML and TK. Furthermore, the complex types, including A2G2F and A2G2FB, were processed in both of tumor tissues and cell lines. These oligosaccharides with differential expression patterns in the tumor tissues resected from patients with CNS lymphomas may be possible oligosaccharide marker candidates to discriminate CNS lymphomas and non-CNS lymphomas.

## Discussion

Molecular alterations characterizing PCNSL have been studied to develop potentially diagnostic and prognostic imaging and biomarkers, proposed as possible targeted therapies [46–49]. There is also great interest in the Bruton’s tyrosine kinase (BTK) inhibitor against DLBCL (ibrutinib), immunomodulatory drugs (lenalidomide), and immunotherapy using anti-programmed cell death 1 (PD-1) monoclonal antibody (nivolumab) [47, 50]. Furthermore, genetic and molecular alterations activating nuclear factor-kappa B (NF-ĸB) signaling are found in most PCNSL together with MYD88 and CD79B mutations [47]. Several studies have suggested a strong correlation between lectin-binding patterns and/or biological significances and functions in various tumors, including DLBCL [24–27], lymphoma [28], and hematopoietic cell lines [28]. However, few studies have been reported on the glycoproteins in CNS lymphomas. Although few clinical specimens were used, the CNS lymphoma tissue-specific oligosaccharides, including GM9, BA-2, A2G0F, BA-1, LewisXa/b-BA-2, Ga/b-BA-2, A2G2, A3G3, and A2G1 (3)(6)F, were isolated, whereas there could have been contaminating blood cells and/or macrophages from the tumor microenvironments. Thus, further studies should be performed carefully in a number of purified tumor specimens.

A2G2F was increased in glioblastoma tissues and glioma cell lines, while being less than 0.1% in normal brain tissue [51]. In the present study, A2G2F was detected in neutral and sialylated sugar chains as 2.19–5.0% but not in neutral sugar chains in the cell lines, which had a slight change in the MTX-resistant cells compared to the control cells as 0.92–1.08-fold (Fig. 2g, h). In sialylated sugar chains, A2G2F was detected as 11.67–44.02% with decreased levels in the MTX-resistant cells compared to the control cells as 0.76–0.81-fold (Fig. 3b, c). Besides, in the analysis for the CNS lymphoma tissues, average 6.59% (range: 2.77–14.54%) of A2G2F was detected in the four clinical specimens (Fig. 4a, b). Therefore, A2G2F in CNS lymphoma tissues and cell lines is much than normal brain tissue, whereas sialylated A2G2F in the MTX-resistant PCNSL-derived cells is slightly decreased compared to the control cells, which is consistent with glioma cell lines, glioblastoma tissues, PCNSL-derived cell lines, CNS lymphoma tissues, and non-CNS lymphoma cells. In glioblastoma and/or glioma, *Lens culinaris* agglutinin (LCA)-lectin binding to A2G2F inhibits cell proliferation of glioma through induction of apoptosis [51]. Therefore, A2G2F may also provide a useful marker candidate and a hint for diagnosis and development for target therapy in CNS lymphoma, as well as glioma/glioblastoma.

All of the four CNS lymphoma specimens and cell lines, including CNS lymphoma and non-CNS lymphoma, used in the study were human immunodeficiency virus (HIV)-negative, except for HBL1 and OYB as no valid information for HIV (Additional file 1: Table S1). PCNSL-derived HKBML and non-CNS lymphoma-derived Raji are Epstein-Barr virus (EBV)-positive and PCNSL-derived TK is EBV-negative (Additional file 1: Table S1). However, the expression patterns of *N*-oligosaccharides in HKBML were similar to that in TK than Raji, in addition to differential expression with acquired MTX-resistance in neutral and sialylated sugar chains (Fig. 2g, h), and sialylated sugar chains (Fig. 3b, c). While, the EBV infection in OYB cells was unknown (Additional file 1: Table S1). Our previous study has clarified that EBV-positive PCNSLs are counted by 20% [52]. However, all of the four clinical specimens examined in the study were EBV-negative. Therefore, whether EBV-positive PCNSLs make a change to their oligopatterns should await future studies.

## Conclusions

In this study, we demonstrated that the expression of specific sialylated *N*-linked oligosaccharides, including A2G2F and A2G2FB, in the MTX-resistant cells slightly decreased compared to the corresponding control cells. Similarly, the decreased expression of sialylated A2G2F and A2G2FB seemed to correlate with poor prognoses of the CNS lymphoma patients, despite the small sample number. Therefore, the differential expression and patterns of surface glycans on CNS lymphomas make it possible to escape the cell–cell recognition by immune cells, thereby, MTX-resistant malignant CNS lymphoma cells could re-grow. In conclusion, the above-mentioned oligosaccharides may be promising oligosaccharide marker candidates to recognize MTX-resistant cells, and primary and secondary CNS lymphomas, which may be useful for diagnosis marker development and/or applied molecular targeted therapies for CNS lymphomas.

## Supplementary information

**Additional file 1: Table S1.** Clinical information of cell lines and CNS lymphoma specimen.

**Additional file 2: Figure S1.** Workflow for isolation and characterization of *N*-linked oligosaccharide from lymphoma cells and central nervous system (CNS) lymphoma clinical specimens. (**A**) Construction of methotrexate (MTX)-resistant lymphoma cells. (i) HKBML and TK as primary central nervous system lymphoma (PCNSL) cells, and RAJI, A4/FUK, HBL1, and OYB as non-CNS lymphomas were used. (ii) HKBML, TK, and RAJI were modified into MTX-resistant cells. (iii) Primary and secondary CNS lymphoma specimens were also used. (**B**) Schematic representation of high performance liquid chromatography (HPLC) for neutral sugar chains and sialylated sugar chains derived from lymphoma cells including PCNSL and non-CNS lymphoma, and CNS lymphoma clinical specimens.

**Additional file 3: Figure S2.** Expression analysis of neutral and sialyl sugar chains with high performance liquid chromatography for the *N*-linked oligosaccharide patterns in human methotrexate (MTX)-resistant lymphoma cells. (**A**) Diethylaminoethyl cellulose (DEAE-C) ion-exchange chromatography for the *N*-linked oligosaccharides in human MTX-resistant lymphoma cells and the corresponding non-resistant cells. Neutral and sialyl sugar chains in HKBML (left), TK (center), and RAJI (right) were detected by S0–S4 (0–4× sialyl sugar chains, respectively). Fractions eluting at retention times of S0 were further analyzed by normal-phase high performance liquid chromatography (HPLC). Arrow heads left-sided at S1 peak indicate *N*-oligosaccharides coupling with an unknown acidic group. Arrows left-sided at S2 peak indicate *N*-mono-sulfated oligosaccharides. S0–S4, numbers coupled with sialic acids or debris. (**B–G**) The peaks representing neutral sugar chains (**B, D, and F**), and neutral and sialyl sugar chains (**C, E, and G**) were detected in HKBML (**B and C**), TK (**D and E**), and RAJI (**F and G**). Fractions eluting at retention times of M4-M9 were collected and analyzed with normal-phase HPLC. The peak numbers refer to the oligosaccharide structures in Additional file 5**: Figure S4**. MU_STD; mannose unit standard including M2B, M3B, M4B, M5A, M6B, M7A, M8A, and M9A, MTX; methotrexate, S0; non-sialyl sugar chain, Neu; neuraminidase-treated sugar chains.

**Additional file 4: Figure S3.** Expression analysis of neutral and sialyl sugar chains with high performance liquid chromatography for the *N*-linked oligosaccharide patterns in human methotrexate (MTX)-resistant lymphoma cells. (**A**) Diethylaminoethyl cellulose (DEAE-C) ion-exchange chromatography for the *N*-linked oligosaccharides in human lymphoma cells. Neutral and sialyl sugar were detected by S0–S4 (0–4× sialyl sugar chains, respectively). Fractions eluting at retention times of S0 were further analyzed by normal-phase high performance liquid chromatography (HPLC). Arrow heads left-sided at S1 peak indicate *N*-oligosaccharides coupling with an unknown acidic group or debris. Arrows left-sided at S2 peak indicate *N*-mono-sulfated oligosaccharides. S0–S4, numbers coupled with sialic acids. (**B–C**) The peaks representing neutral sugar chains (**B**), and neutral and sialyl sugar chains (**C**) were detected in OYB (green), HBL1 (blue), and A4/FUK (red) cells. Fractions eluting at retention times of M4–M9 were collected and analyzed with normal-phase HPLC. The peak numbers refer to the oligosaccharide structures in Additional file 5**: Figure S4**. MU_STD; mannose unit standard including M2B, M3B, M4B, M5A, M6B, M7A, M8A, and M9A, S0; non-sialyl sugar chain, Neu; neuraminidase-treated sugar chains.

**Additional file 5: Figure S4.** Structures of PA-oligosaccharides. High mannose type oligosaccharides; GM9, M9A, M8A, M7A, M7B, M6B, and M5A. Processing oligosaccharide; M4B. Complex type oligosaccharides; BA-2 (A2G0FB), A2G0F, BA-1, LewisXa/b-BA-2, Ga/b-BA2, G2BA-2 (A2G2FB), A2G2, A2G2F, A3G3, A3G3F, A4G4F, and A2G1 (3)(6)F. GlcNAc; *N*-acetylglucosamine, Man; mannose, Gal; galactose, Glu; glucose, Fuc; fucose. PA; pyridylamination. The nomenclature of structures is shown as follows: An (*n* = 2–4) indicates the number of antennae linked to the bi−/tri−/tetra-mannosyl core, Gn (*n* = 0–4) is the number of galactose residues attached to the nonreducing ends, F indicates a fucosylation core, and B refers to bisecting *N*-acetylglucosamine.

**Additional file 6: Figure S5.** Differential expression of neutral and sialyl sugar chains in normal-phase HPLC analysis of the *N*-linked oligosaccharide patterns of human defuse large B cell lymphoma (DLBCL) cells. (A–B) Neutral (**A**) and sialyl (**B**) sugar chains were detected in non-CNS lymphomas, including A4/FUK, HBL1, and OYB. Intensities were evaluated with the percent (%) area of peaks in each fraction eluting at retention times of M4, M5, M6, M7, M8, and M9. The peak numbers refer to the oligosaccharide structures in Additional file 5: Figure S4. (**C**) Summary for expression changes of neutral and sialyl sugar chains among A4/FUK, HBL1, and OYB as non-CNS lymphomas. Color configuration indicates high (red) to low (green). S0: non-sialyl sugar chain, Neu: Neuraminidase-treated sugar chains. (**D**) Percent area (%) of peaks in each fraction eluting at retention times of sialyl sugar chains form A4/FUK (red), HBL1 (blue), and OYB (green). (**E**) Summary of percent area of sialyl sugar chains among A4/FUK, HBL1, and OYB. Color configuration indicates high (red) to low (black). NA, not applicable.

## Data Availability

The data analyzed during the current study are available from the corresponding author on reasonable request.
