# Supplementary material for: Differential expression of N-linked oligosaccharides in methotrexate-resistant primary central nervous system lymphoma cells
Source: BMC Cancer. 2019 Sep 11;19:910. doi: 10.1186/s12885-019-6129-8 (PMC6739943; doi:10.1186/s12885-019-6129-8)
Supplement: Supplementary file 1 — Additional file 1: Table S1. Clinical information of cell lines and CNS lymphoma specimen. [file 12885_2019_6129_MOESM1_ESM.docx]

**Table S1. Clinical information of cell lines and CNS lymphoma specimens**

|  | **Age (years)** | **Gender** | **Primary/secondary** | **Histology** | **HIV^1^** | **EBV^2^** |
| --- | --- | --- | --- | --- | --- | --- |
| **(Cell lines)** |  |  |  |  |  |  |
| **HKBML** | **NA^3^** | **Male** |  | **PCNSL^4^** | **(-)** | **(+)** |
| **TK** | **22** | **Male** |  | **PCNSL, ABC-DLBCL^5^** | **(-)** | **(-)** |
| **Raji** | **11** | **Male** |  | **Burkitt's lymphoma** | **(-)** | **(+)** |
| **A4/Fuk** | **52** | **Female** |  | **IgM kappa-producing B-cell lymphoma** | **(-)** | **(-)** |
| **HBL1** | **65** | **Male** |  | **ABC-DLBCL** | **NA** | **(-)** |
| **OYB** | **NA** | **NA** |  | **ABC-DLBCL** | **NA** | **ND^6^** |
|  |  |  |  |  |  |  |
| **(CNS lymphoma specimens)** | |  |  |  |  |  |
| **Sample 1** | **57** | **Male** | **PCNSL** | **DLBCL** | **(-)** | **(-)** |
| **Sample 2** | **58** | **Male** | **PCNSL** | **DLBCL** | **(-)** | **(-)** |
| **Sample 3** | **85** | **Female** | **PCNSL** | **DLBCL** | **(-)** | **(-)** |
| **Sample 4** | **73** | **Male** | **Secondary CNS lymphoma** | **DLBCL** | **(-)** | **(-)** |
|  |  |  |  |  |  |  |

**Note: (+); positive, (-); negative, ^1^HIV; human immunodeficiency virus, ^2^EBV; Epstein-Barr virus, ^3^NA; not applicable, ^4^PCNSL; primary central nervous system lymphoma, ^5^ABC; activated B-cell-like subtype, ^6^ND; not determined.**
