# Supplementary figures and images for: Differential expression of N-linked oligosaccharides in methotrexate-resistant primary central nervous system lymphoma cells
Source: BMC Cancer. 2019 Sep 11;19:910. doi: 10.1186/s12885-019-6129-8 (PMC6739943; doi:10.1186/s12885-019-6129-8)

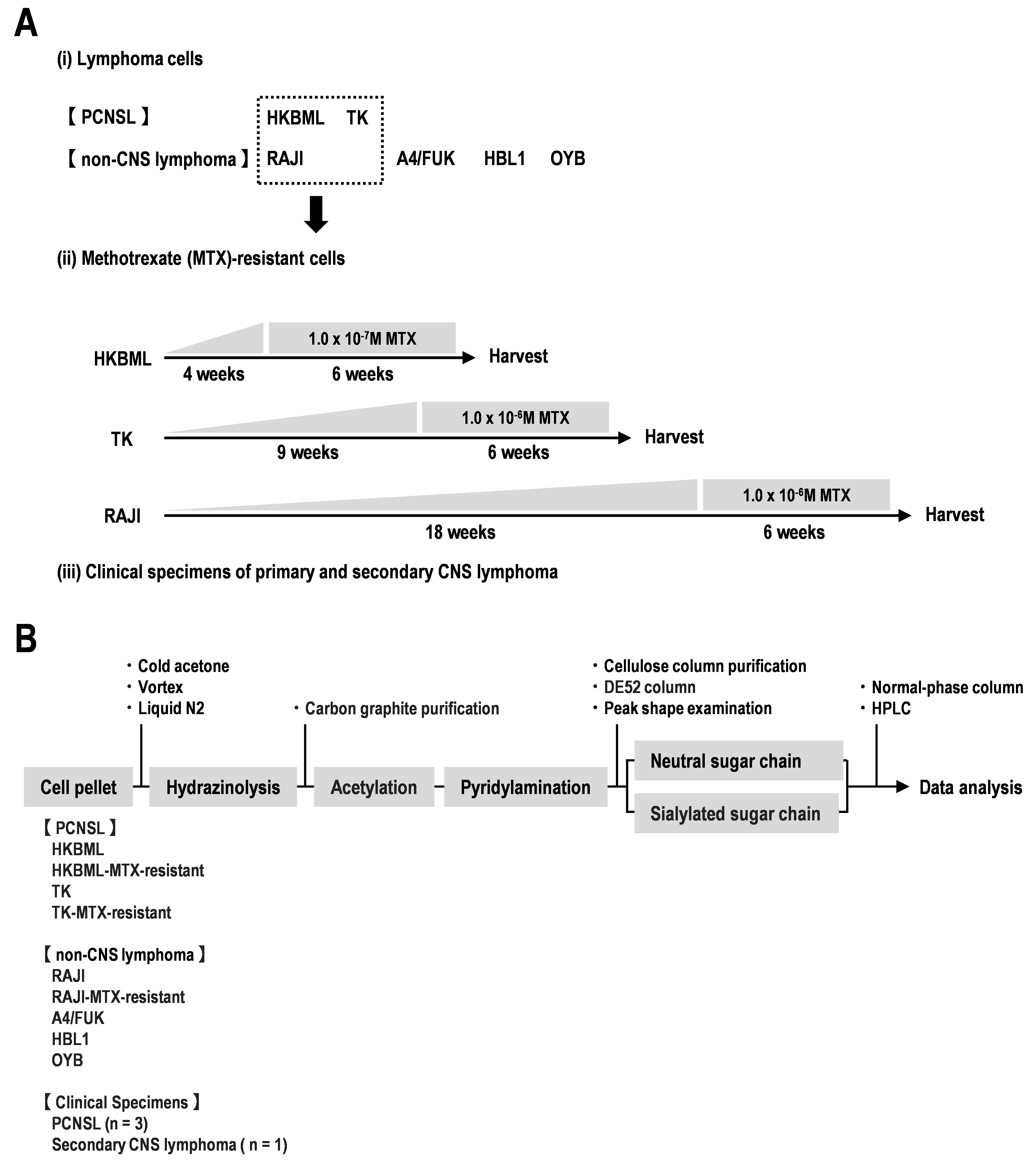

Supplement: Supplementary file 2 — Additional file 2: Figure S1. Workflow for isolation and characterization of N-linked oligosaccharide from lymphoma cells and central nervous system (CNS) lymphoma clinical specimens. (A) Construction of methotrexate (MTX)-resistant lymphoma cells. (i) HKBML and TK as primary central nervous system lymphoma (PCNSL) cells, and RAJI, A4/FUK, HBL1, and OYB as non-CNS lymphomas were used. (ii) HKBML, TK, and RAJI were modified into MTX-resistant cells. (iii) Primary and secondary CNS lymphoma specimens were also used. (B) Schematic representation of high performance liquid chromatography (HPLC) for neutral sugar chains and sialylated sugar chains derived from lymphoma cells including PCNSL and non-CNS lymphoma, and CNS lymphoma clinical specimens. [file 12885_2019_6129_MOESM2_ESM.tif]

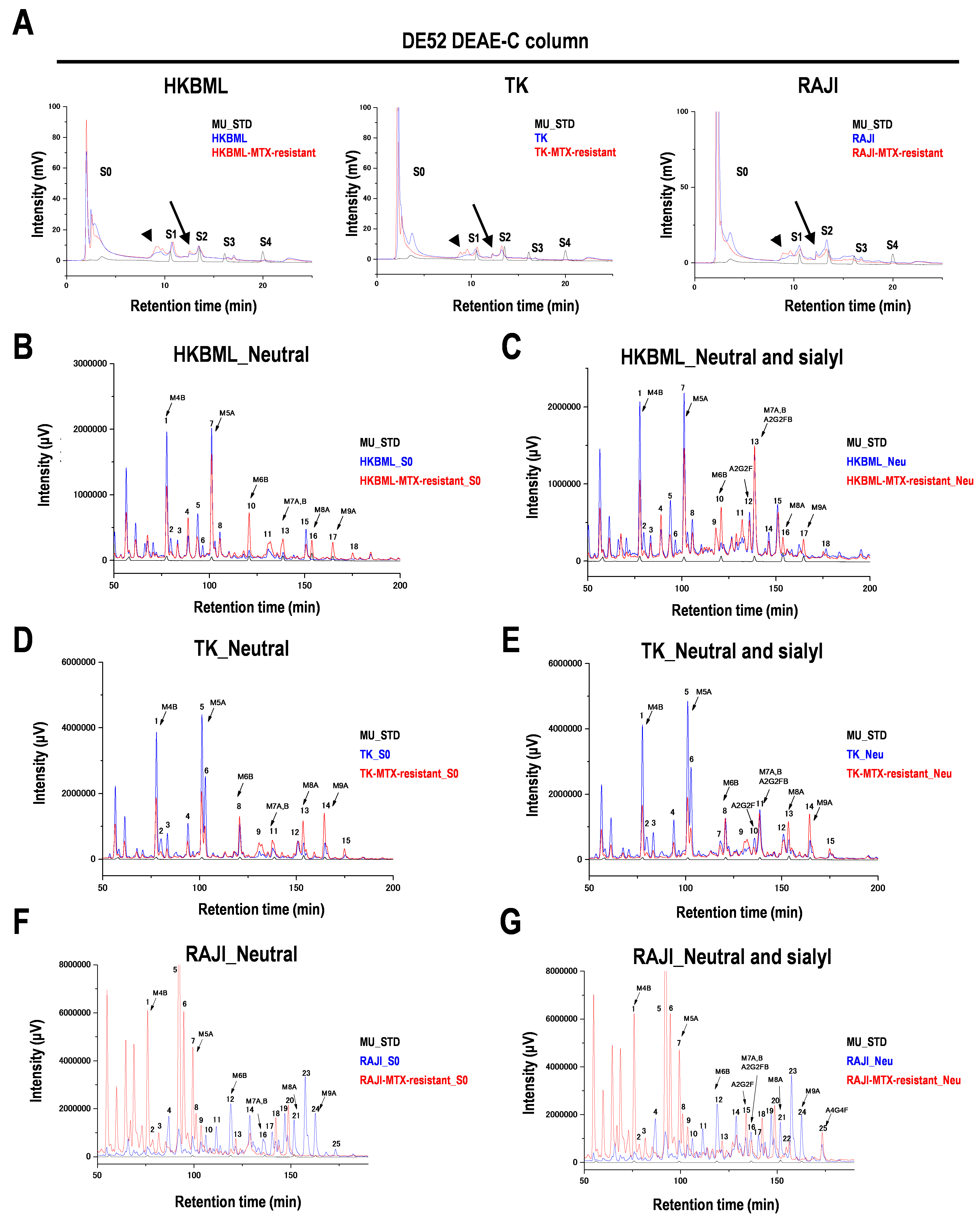

Supplement: Supplementary file 3 — Additional file 3: Figure S2. Expression analysis of neutral and sialyl sugar chains with high performance liquid chromatography for the N-linked oligosaccharide patterns in human methotrexate (MTX)-resistant lymphoma cells. (A) Diethylaminoethyl cellulose (DEAE-C) ion-exchange chromatography for the N-linked oligosaccharides in human MTX-resistant lymphoma cells and the corresponding non-resistant cells. Neutral and sialyl sugar chains in HKBML (left), TK (center), and RAJI (right) were detected by S0–S4 (0–4× sialyl sugar chains, respectively). Fractions eluting at retention times of S0 were further analyzed by normal-phase high performance liquid chromatography (HPLC). Arrow heads left-sided at S1 peak indicate N-oligosaccharides coupling with an unknown acidic group. Arrows left-sided at S2 peak indicate N-mono-sulfated oligosaccharides. S0–S4, numbers coupled with sialic acids or debris. (B–G) The peaks representing neutral sugar chains (B, D, and F), and neutral and sialyl sugar chains (C, E, and G) were detected in HKBML (B and C), TK (D and E), and RAJI (F and G). Fractions eluting at retention times of M4-M9 were collected and analyzed with normal-phase HPLC. The peak numbers refer to the oligosaccharide structures in Additional file 5: Figure S4. MU_STD; mannose unit standard including M2B, M3B, M4B, M5A, M6B, M7A, M8A, and M9A, MTX; methotrexate, S0; non-sialyl sugar chain, Neu; neuraminidase-treated sugar chains. [file 12885_2019_6129_MOESM3_ESM.tif]

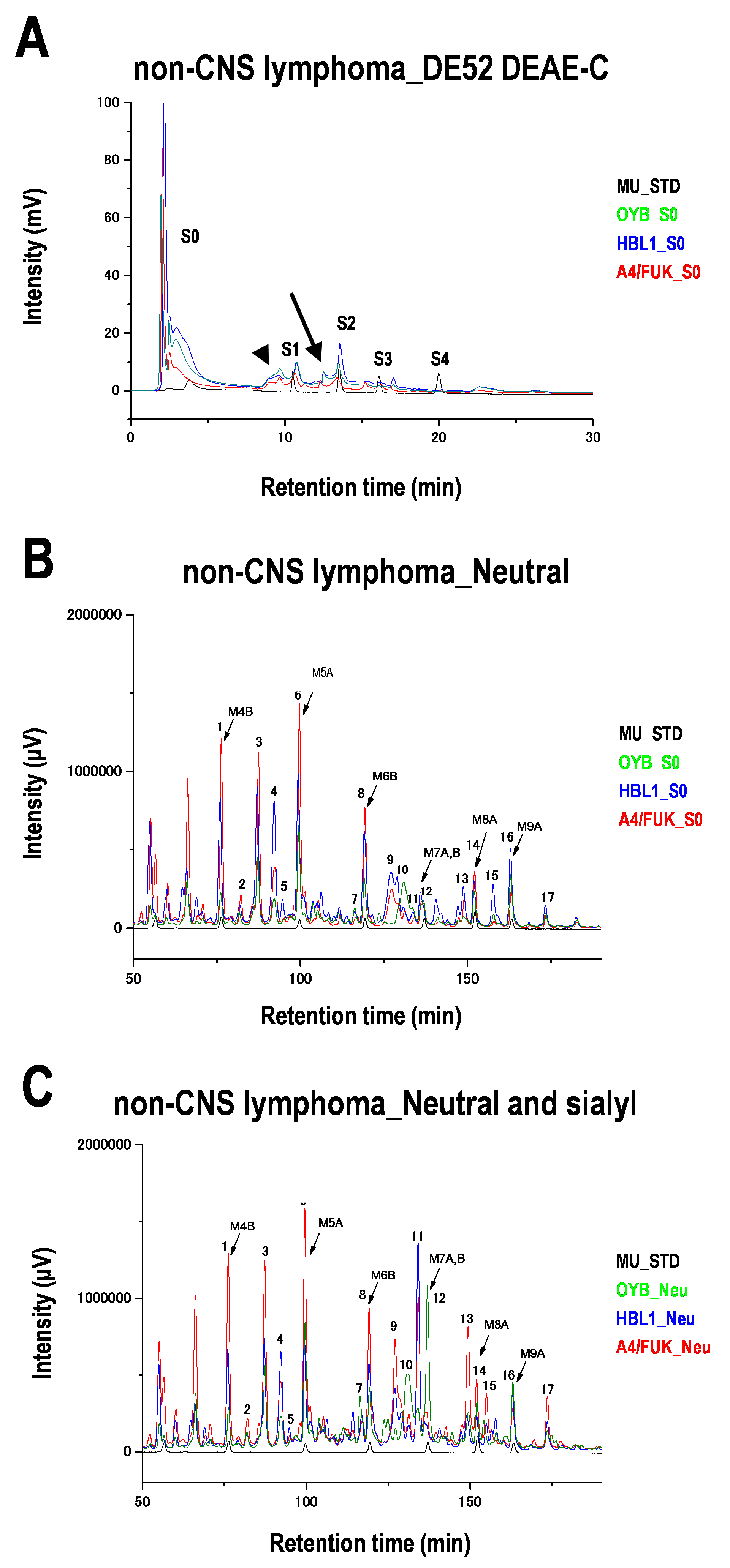

Supplement: Supplementary file 4 — Additional file 4: Figure S3. Expression analysis of neutral and sialyl sugar chains with high performance liquid chromatography for the N-linked oligosaccharide patterns in human methotrexate (MTX)-resistant lymphoma cells. (A) Diethylaminoethyl cellulose (DEAE-C) ion-exchange chromatography for the N-linked oligosaccharides in human lymphoma cells. Neutral and sialyl sugar were detected by S0–S4 (0–4× sialyl sugar chains, respectively). Fractions eluting at retention times of S0 were further analyzed by normal-phase high performance liquid chromatography (HPLC). Arrow heads left-sided at S1 peak indicate N-oligosaccharides coupling with an unknown acidic group or debris. Arrows left-sided at S2 peak indicate N-mono-sulfated oligosaccharides. S0–S4, numbers coupled with sialic acids. (B–C) The peaks representing neutral sugar chains (B), and neutral and sialyl sugar chains (C) were detected in OYB (green), HBL1 (blue), and A4/FUK (red) cells. Fractions eluting at retention times of M4–M9 were collected and analyzed with normal-phase HPLC. The peak numbers refer to the oligosaccharide structures in Additional file 5: Figure S4. MU_STD; mannose unit standard including M2B, M3B, M4B, M5A, M6B, M7A, M8A, and M9A, S0; non-sialyl sugar chain, Neu; neuraminidase-treated sugar chains. [file 12885_2019_6129_MOESM4_ESM.tif]

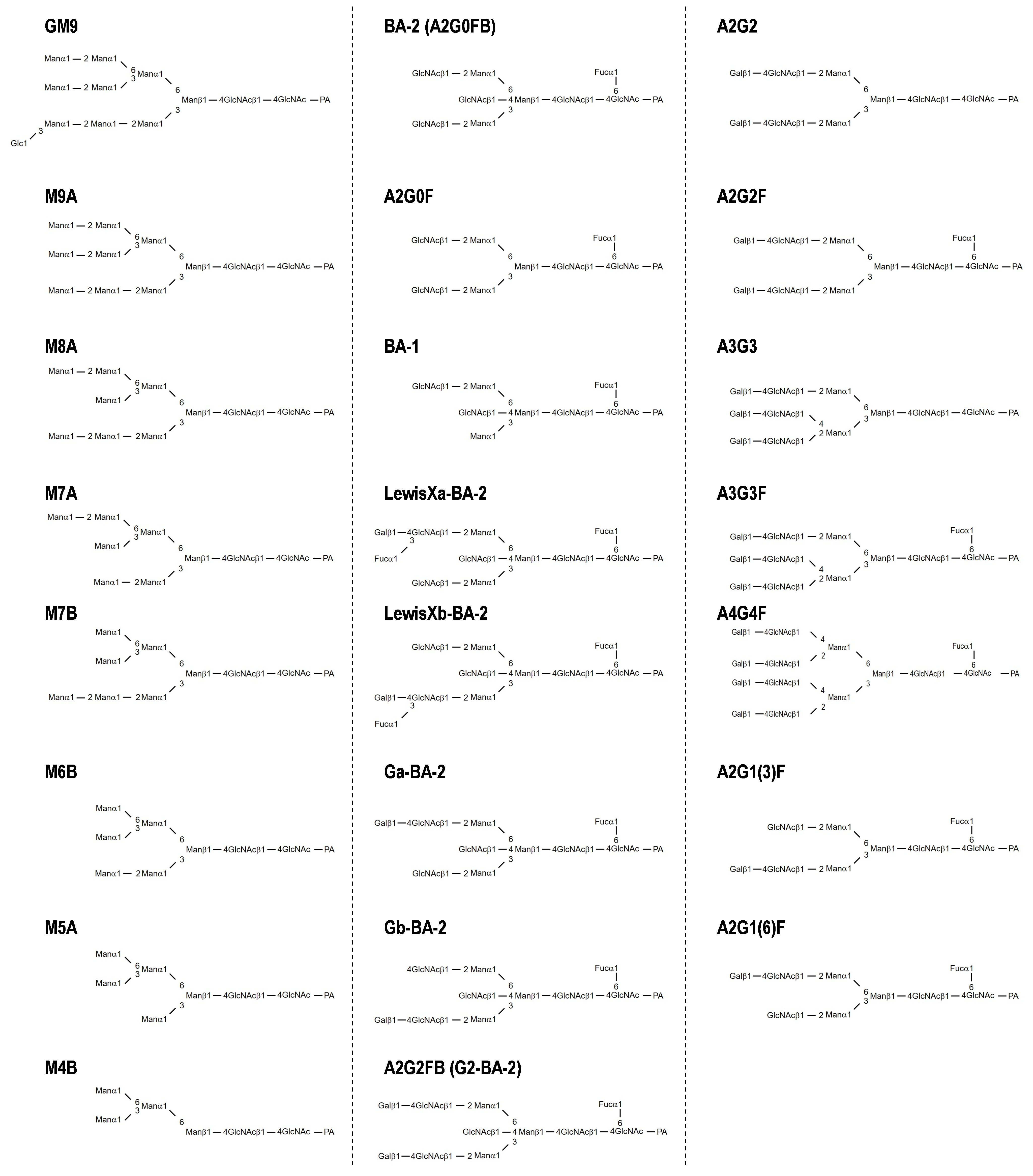

Supplement: Supplementary file 5 — Additional file 5: Figure S4. Structures of PA-oligosaccharides. High mannose type oligosaccharides; GM9, M9A, M8A, M7A, M7B, M6B, and M5A. Processing oligosaccharide; M4B. Complex type oligosaccharides; BA-2 (A2G0FB), A2G0F, BA-1, LewisXa/b-BA-2, Ga/b-BA2, G2BA-2 (A2G2FB), A2G2, A2G2F, A3G3, A3G3F, A4G4F, and A2G1 (3)(6)F. GlcNAc; N-acetylglucosamine, Man; mannose, Gal; galactose, Glu; glucose, Fuc; fucose. PA; pyridylamination. The nomenclature of structures is shown as follows: An (n = 2–4) indicates the number of antennae linked to the bi−/tri−/tetra-mannosyl core, Gn (n = 0–4) is the number of galactose residues attached to the nonreducing ends, F indicates a fucosylation core, and B refers to bisecting N-acetylglucosamine. [file 12885_2019_6129_MOESM5_ESM.tif]

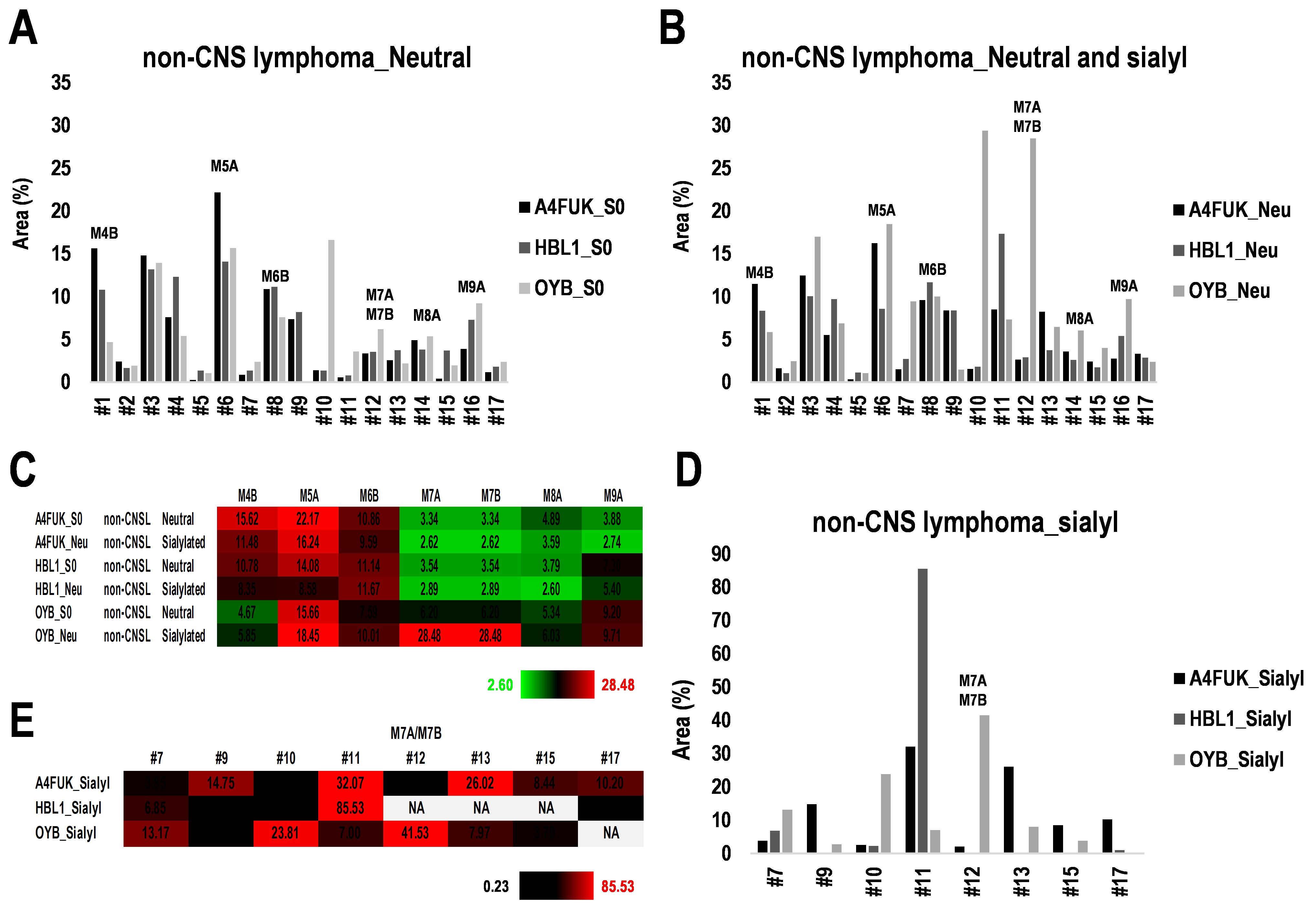

Supplement: Supplementary file 6 — Additional file 6: Figure S5. Differential expression of neutral and sialyl sugar chains in normal-phase HPLC analysis of the N-linked oligosaccharide patterns of human defuse large B cell lymphoma (DLBCL) cells. (A–B) Neutral (A) and sialyl (B) sugar chains were detected in non-CNS lymphomas, including A4/FUK, HBL1, and OYB. Intensities were evaluated with the percent (%) area of peaks in each fraction eluting at retention times of M4, M5, M6, M7, M8, and M9. The peak numbers refer to the oligosaccharide structures in Additional file 5: Figure S4. (C) Summary for expression changes of neutral and sialyl sugar chains among A4/FUK, HBL1, and OYB as non-CNS lymphomas. Color configuration indicates high (red) to low (green). S0: non-sialyl sugar chain, Neu: Neuraminidase-treated sugar chains. (D) Percent area (%) of peaks in each fraction eluting at retention times of sialyl sugar chains form A4/FUK (red), HBL1 (blue), and OYB (green). (E) Summary of percent area of sialyl sugar chains among A4/FUK, HBL1, and OYB. Color configuration indicates high (red) to low (black). NA, not applicable. [file 12885_2019_6129_MOESM6_ESM.tif]
